# Use, trustworthiness and willingness to self-monitor health and personal wellness: a UK population survey

**DOI:** 10.1186/s13690-026-02035-6

**Published:** 2026-08-13

**Authors:** Kevin J. Munro, Lucy Ferrie, Sarah A. Rhodes, Gabrielle H. Saunders, Gareth Smith, Adele M. Goman

**Affiliations:** 1 Manchester Centre for Audiology and Deafness, University of Manchester, Manchester, M13 9PL UK; 2 Centre for Biostatistics, Division of Population Health, Health Services Research & Primary Care, School of Health Sciences, The University of Manchester, Manchester, M13 9PL UK; 3 Department of Audiology, Mid and South Essex Hospital NHS Foundation Trust, Southend Hospital Site, Prittlewell Chase, Westcliff on Sea, SS0 0RY UK; 4 School of Psychology, University of Leeds, Leeds, LS2 9JT UK

**Keywords:** Self-monitoring, Public attitudes, Online survey, Experiences, Willingness, Trust, Health and personal wellness

## Abstract

**Background:**

Public opinion about self-monitoring of health and personal wellness is important but largely unknown. This study investigated public attitudes towards self-monitoring of health and personal wellness.

**Methods:**

Four thousand two hundred seventy UK adults selected to produce a nationally representative sample (by age, gender, ethnicity, social grade/socioeconomic group, geographic region) completed an online survey. Self-monitoring questions addressed experiences (use, motivation, trust, actions taken), benefits, problems, willingness (to use, to have results sent to GP) and the preferred action if a problem is identified. Questions concerned 12 physiologic and behavioural health aspects (blood pressure, blood sugar, body composition, cholesterol, diet, eyesight, fitness/exercise, hearing, menstrual cycles/periods, mental and cognitive health, pulse/heart rate, and skin conditions).

**Results:**

Most respondents had self-monitored (79%) and were willing, if recommended by the NHS, to self-monitor (86%) at least one health aspect. The proportion of respondents who had self-monitored was similar for health and personal wellness; however, the proportion willing to self-monitor was higher for health than personal wellness. Self-monitoring was higher among younger adults and those with more years of education. Most (70–94%) respondents trusted the results; 78% were willing to have results sent automatically to their GP. If a problem were identified, discussing the results with a GP was a common and preferred action, but 14–38% of respondents (depending on health aspect) took no action. Males, older adults, those living in areas of high deprivation, and those with lower education were less likely to be willing to monitor their health. Main benefits were enhanced awareness of health, greater control, easier access to information, more balanced engagement with providers, and psychological support. Main problems included anxiety, obsessive testing, low self-efficacy for monitoring, poor motivation, post-monitoring inaction, misinterpretation of results, and technical concerns.

**Conclusions:**

A high proportion of the UK public use/are willing to use and trust self-monitoring of health and wellness. It has the potential to transform care through proactive remote health management and preventative action. However, the findings also highlight socioeconomic differences in self-monitoring. Future research is needed to address how engagement of self-monitoring can be enhanced equitably and used effectively alongside clinical care to promote better health.

**Trial registration:**

https://osf.io/96mzt.

**Supplementary Information:**

The online version contains supplementary material available at https://doi.org/10.1186/s13690-026-02035-6.

**Table:** 

Text box 1. Contributions to the literature	
• The UK Government has outlined aspirations for health monitoring technologies to be part of routine care to support proactive health monitoring and preventative action.	
• Public opinion about self-monitoring is important because this has the potential to change how the public will engage with future NHS initiatives on self-monitoring of health.	
• Our study suggests the UK public use, trust, and are willing to self-monitor their health and personal wellness, and have results sent to their GP.	
• Socioeconomic differences in willingness to self-monitor were found highlighting the need to address how engagement in self-monitoring can be enhanced equitably and used effectively alongside clinical care.	

## Background

The revolution in interest in new technologies and adoption of personal health self-monitoring has the potential to transform health care [1]. Health can be tracked using tools such as wearable technologies, biosensors, smart medical devices, apps and over-the-counter diagnostic kits [2]. The motivation for self-monitoring of health may be for medical reasons (e.g., blood glucose monitoring for managing diabetes), or for wellness purposes (e.g., monitoring exercise for fitness). Reported benefits to public health include:

i. Increased self-awareness and promotion of preventative behaviours,
ii. early detection of health issues,
iii. enhanced self-efficacy and management of chronic conditions,
iv. lifestyle changes,
v. improved treatment adherence,
vi. reduced pressure on healthcare systems,
vii. better communication with healthcare providers, and.
viii. improved outcomes [3, 4].

The UK government has expressed considerable interest in self-monitoring of healt [5] and there is an appetite amongst the public to use technology to take greater responsibility for their health [6]. While users generally show positive attitudes toward health monitoring technologies, concerns have been expressed over accuracy, cost, and burden (to the public and healthcare system) [1]. There are also concerns about the current market and regulatory oversight for some direct-to-consumer tests [7, 8]. Healthcare providers have recognised the potential benefits of self-monitoring for patient health [9] but also concerns (e.g. workload) [10].

Public opinion about self-monitoring of health and wellness is important because, by nature, self-monitoring requires voluntary active engagement if future NHS initiatives including preventative approaches are to be successful. Previous UK survey [6, 11] have examined use of self-monitoring technology, but not whether the public trusts the results or what actions are taken (or preferred) following self-monitoring. Therefore, the aim of the study was to investigate attitudes and experiences towards self-monitoring of health and wellness over a broad range of health aspects. We achieved this by undertaking a cross-sectional survey of UK adults. The survey included questions related to willingness to pay and share data, within the context of the NHS.

## Methods

### Respondents

Respondents were adults living in the UK selected by YouGov PLC UK (an internet-based research service) from members signed up to the survey platform. A target sample size of 4000 allowed us to estimate key prevalences to a precision of ± 1.5%.

### Online survey

Nine survey questions (Supplementary Table S1) were embedded into a YouGov survey completed between 31^st^ July and August 13^th^, 2025. The questions were developed by the study team and addressed:

1. Self-monitoring experience, trust in results, actions taken and whether monitoring was recommended by a health professional (questions 1–4).
2. Willingness to self-monitor, and, of those willing, the proportion willing to have results automatically sent to their GP or other health professional, willingness to pay for self-monitoring (assuming a similar cost to an NHS prescription) and preferred next steps if a problem were identified (questions 7–9).
3. Main benefits of, and problems with, self-monitoring (questions 5–6).

For (a) and (b), participants indicated their experience and willingness to self-monitor a range of medical health and wellness health aspects: blood pressure, blood sugar, body composition, cholesterol, diet, eyesight, fitness/exercise, hearing, menstrual cycles/periods, mental and cognitive health, pulse/heart rate, and skin conditions. An ‘Other’ option was available for respondents to indicate if they had monitored any other health aspect not listed. For (c), participants were asked two open ended questions: one on benefit of self-monitoring and one on problems experienced when self-monitoring.

### Patient and public involvement

Twelve members of the public (age range: 20–69 years) provided feedback on the draft survey. While none thought it was burdensome or time consuming, they recommended that we provide a definition of ‘self-monitoring’, examples of what this might entail, and to add additional health conditions to our list (e.g., skin conditions and blood glucose).

### Procedure

YouGov invited individuals signed up to their platform to complete their weekly survey, incorporating our 9 questions, via a link in an email. YouGov set quotas and monitored completion rates by key demographics (age, gender, ethnicity, geographical region, and social grade/socio-economic group) using information held on their panel member database. Once the desired number of respondents was reached, the survey link became inactive. Post-weighting was then applied by YouGov to ensure the final dataset was representative of the UK population according to the key demographics. Highest level of education, gender, age, and index of multiple deprivation (IMD) data were provided by YouGov from data held on their panel member database.

### Analysis

Analyses were pre-registered on Open Science Framework (https://osf.io/96mzt/overview). YouGov provided summary weighted and unweighted (raw) data for 4341 respondents. There were no major differences between the weighted and unweighted data, so unweighted data were used for all analyses. Two authors independently reviewed open-ended responses and noted any seemingly generated by AI (e.g., ChatGPT). All data from these respondents were removed from analyses (*n* = 71, 1.64%). This resulted in an analytic sample of 4270 respondents.

Statistical analyses were performed using Stata 19.0. Categorial responses were summarised in number and percentages. To explore potential differences between self-monitoring experiences and willingness to monitor for medical and personal wellness purposes the research team selected (post-hoc) two health aspects that commonly relate to physiologic monitoring for medical purposes (blood pressure and blood sugar) and two behavioural health aspects that relate more to personal wellness (fitness/exercise and diet) for comparison. Whilst the reasons for monitoring were not gathered in the present study, similar distinctions in use have been considered previously [12]. Logistic regression analyses examined the relationship between key outcomes: (i) Has self-monitored, (ii) Would be willing to monitor, and (iii) Would be willing to pay for an app or equipment) and explanatory variables (preregistered as likely covariates: health condition, highest level of education, gender, age, and IMD). Multilevel logistic regression with a random effect term for ‘person’ was used for outcomes (i) and (ii). Any respondent answering ‘*I don’t know*’ for outcome (iii) was excluded from analyses as were two participants with missing IMD data. For monitoring of ‘menstrual cycle/periods’ only data from respondents identifying as female were included.

Conclusions remained unchanged after sensitivity analyses that (a) removed IMD and education from analyses in turn due to multicollinearity concerns; (b) reinstated suspicious cases; (c) replaced ‘I don’t know’ responses with a negative response.

We used 95% confidence intervals (CI) throughout. Exact confidence intervals were included for key prevalence statistics.

Open-ended question responses were analysed using inductive content analysis [13] achieved in a two-step process using NVivo 14 [14]. First, the data were reduced by assigning codes to each response. Where multiple concepts were described within a response, multiple codes were assigned. Second, similar codes were grouped together to form categories. Codes within each category were then further grouped into sub-categories, as considered appropriate.

## Results

Respondents were broadly representative of the UK population in age structure and gender (e.g., slightly more females than males), IMD and education (Table 1).

**Table 1:** Summary characteristics of the 4270 survey respondents

	*n* (%)
Total	4,270
Gender	
Male	2,023 (47.4%)
Female	2,247 (52.6%)
Age	
18–24	443 (10.4%)
25–34	722 (16.9%)
35–44	701 (16.4%)
45–54	778 (18.2%)
55–64	652 (15.3%)
65+	974 (22.8%)
Index of Multiple Deprivation (IMD)	
Decile 1 to 3 (most deprived)	1,102 (25.8%)
Decile 4 to 7 (middle)	1,767 (41.4%)
Decile 8 to 10 (least deprived)	1,399 (32.8%)
Highest education level	
Secondary school	815 (19.1%)
Further education but not university	1,593 (37.3%)
Higher education	1,862 (43.6%)

### Self-monitoring experience and willingness to self-monitor

**Fig. 1:**
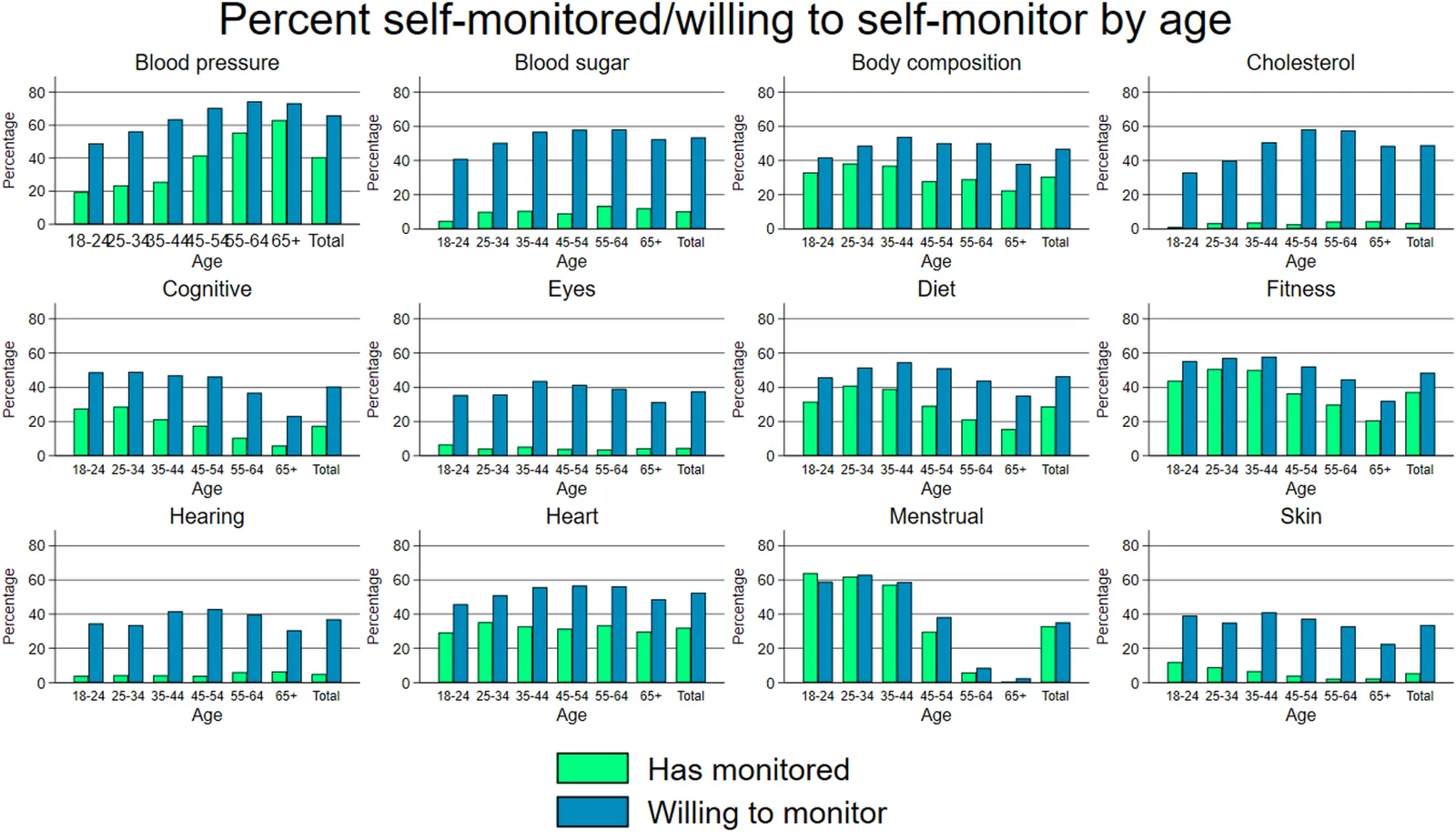
Percentage of respondents who had ever self-monitored (green bars) and were willing to self-monitor (blue bars) each health aspect by age. Top row from left: blood pressure, blood sugar, body composition, cholesterol; middle row from left: mental/cognitive health, eyes/eyesight, diet, fitness/exercise; bottom row from left: hearing, heart rate/pulse, menstrual cycle/periods and skin conditions. A minimum of 3678 were willing to monitor at least one of the 12 aspects of health

Among the 4270 respondents in the analytic sample, 3354 (79%, 95% CI: 77, 80) indicated they had monitored at least one of the 12 listed aspects of health, 2540 (59%) had monitored at least two, 1757 (41%) had monitored at least three, and 1101 (26%) had monitored at least four. 1858 (44%) had monitored at least one of blood pressure and blood sugar compared to 2009 (47%) who had monitored at least one of fitness/exercise and diet. Fig. 1 shows blood pressure was the health aspect with the highest proportion of respondents (41%) who had self-monitored, followed by fitness/exercise (37%). Self-monitoring of blood pressure was more common among older individuals, whereas the converse was true for monitoring of fitness/exercise. The least monitored health aspects were cholesterol (3%) and sensory health (eye/hearing, 5%). Although provided with the opportunity, few respondents (< 2%) indicated they had self-monitored any other health aspect.

**Fig. 2:**
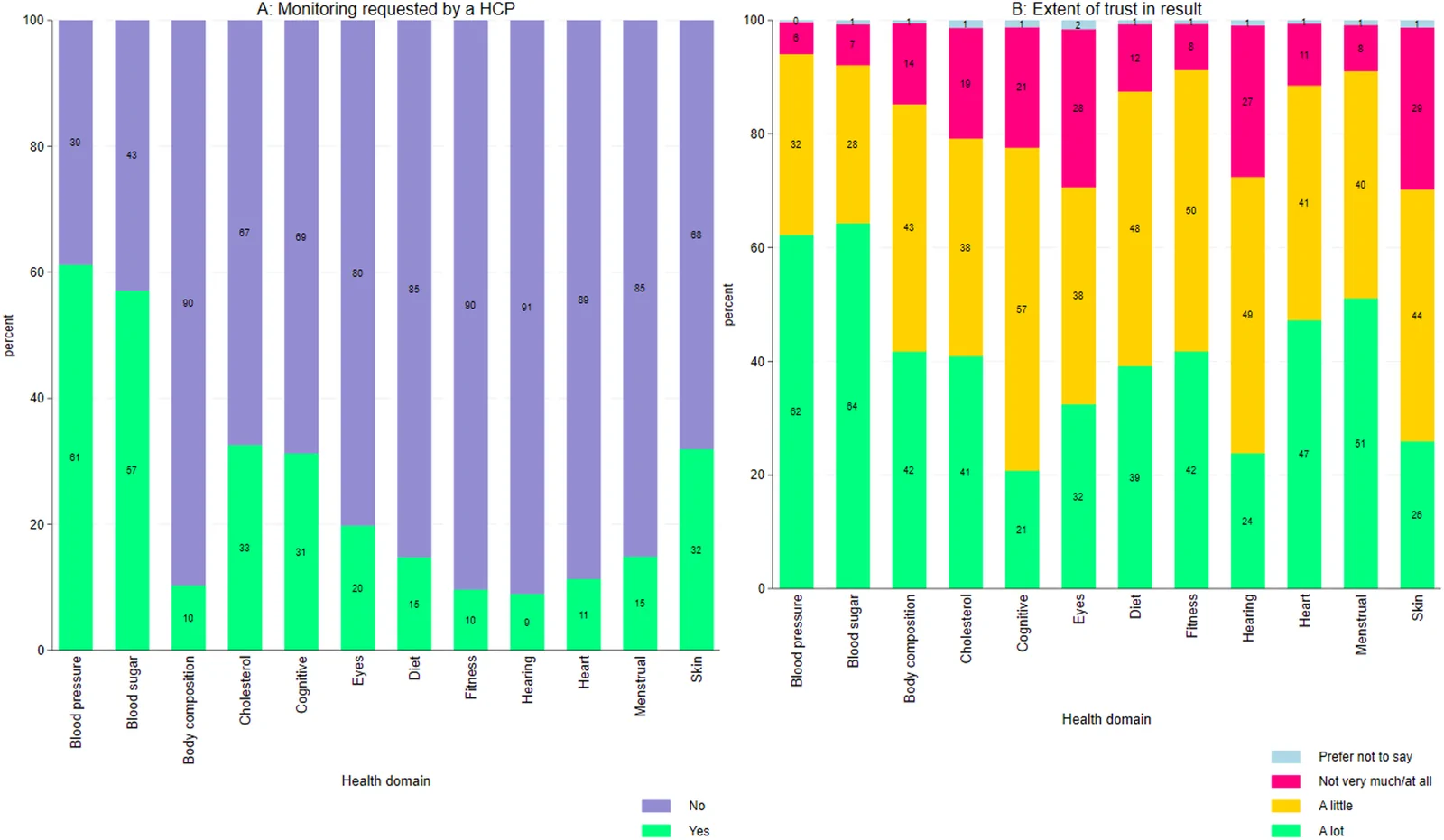
Percentage of respondents who had self-monitored each health aspect following a request by a health professional (panel **A**), and who trusted the results of self-monitoring (panel **B**) by health aspect. The 12 health aspects (from left): blood pressure, blood sugar, body composition, cholesterol, mental/cognitive health, eyes/eyesight, diet, fitness/exercise, hearing, heart rate/pulse, menstrual cycle/periods and skin conditions

Most of the self-monitoring was self-motivated; blood pressure and blood sugar monitoring were most often recommended by a health professional (Fig. 2A). For all health aspects, most respondents indicated they trusted (either a little or a lot) the results (Fig. 2B). Trust was highest for blood pressure (94%) and blood sugar (92%), and lowest for skin conditions (70%) and eyesight (70%). Trust was largely similar when self-monitoring had been recommended by a health professional such as a GP than when it hadn’t, with biggest discrepancies for eyes (82% vs. 70%) hearing (85% vs. 72%) and skin conditions (79% vs. 66%).

If recommended by the NHS, 3678 (86%, 95% CI: 85, 87) were willing to monitor at least one of the 12 health aspects. 3045 (71%) were willing to monitor at least one of blood pressure and blood sugar compared to 2488 (58%) who were willing to monitor at least one of fitness/exercise and diet. Fig. 1 shows that blood pressure had the highest proportion of respondents (66%) willing to self-monitor, followed by blood sugar and pulse/heart rate (both 53%). Health aspects with the lowest proportion of respondents willing to self-monitor were skin conditions (34%), hearing (37%) and eyesight (38%). Among females, willingness to self-monitor menstrual cycles was high across pre-menopausal ages.

Among respondents who had self-monitored, between 15% and 56% (skin conditions and pulse/heart rate, respectively) indicated that monitoring did not show a problem (Supplementary Table S2). Respondents were more likely to indicate that monitoring did not show a problem when this was self-initiated than when it had been recommended by a health professional. The highest discrepancies in monitoring not showing a problem between health professional recommended and self-initiated monitoring were for menstrual (17% vs. 60%), heart (24% vs. 60%) and hearing (5% vs. 40%). Discussing the findings with a GP was the most preferred action if a problem were identified for blood pressure, blood sugar, cholesterol, hearing, mental/cognitive health, menstrual cycle, and skin conditions (Table 2). Between 14 and 38% of respondents reported taking no action following identification of a problem, with the highest rates of inaction being for pulse/heart rate (38%) and hearing (34%). When monitoring had been recommended by a health professional, there was a lower proportion of respondents saying that ‘no action’ was taken than when it hadn’t been recommended by a health professional. Largest differences in inactivity between monitoring recommended by a health professional versus not were seen for blood pressure (9% vs. 30%), menstrual (12% vs. 32%) and hearing (11% vs. 37%).

**Table 2:** Actions taken by respondents after self-monitoring indicated a problem or concern among respondents who had self-monitored the health aspect

	Discussed with GP	Discussed with another HP	Sought advice online	Changed my lifestyle	Did not take action	Don’t know	Prefer not to say
Blood pressure (*n* = 1011)	616 (61%)	123 (12%)	68 (7%)	136 (13%)	152 (15%)	11 (1%)	15 (1%)
Blood sugar (*n* = 285)	126 (44%)	62 (22%)	28 (10%)	72 (25%)	39 (14%)	11 (4%)	8 (3%)
Body composition (*n* = 849)	114 (13%)	57 (7%)	90 (11%)	467 (55%)	195 (23%)	15 (2%)	19 (2%)
Cholesterol (*n* = 109)	63 (58%)	11 (10%)	7 (6%)	28 (26%)	16 (15%)	4 (4%)	0 (0%)
Diet (*n* = 791)	73 (9%)	52 (7%)	120 (15%)	487 (62%)	120 (15%)	30 (4%)	20 (3%)
Eyesight (*n* = 133)	29 (22%)	61 (46%)	8 (6%)	16 (12%)	29 (22%)	6 (5%)	2 (2%)
Fitness/ exercise (*n* = 771)	48 (6%)	34 (4%)	111 (14%)	413 (54%)	204 (26%)	22 (3%)	22 (3%)
Hearing (*n* = 134)	48 (36%)	29 (22%)	15 (11%)	6 (4%)	45 (34%)	1 (1%)	3 (2%)
Mental/ cognitive health (*n* = 582)	246 (42%)	112 (19%)	91 (16%)	134 (23%)	100 (17%)	24 (4%)	19 (3%)
Menstrual cycle (*n* = 342)	162 (47%)	16 (5%)	58 (17%)	42 (12%)	91 (27%)	7 (2%)	12 (4%)
Pulse/heart rate (*n* = 601)	219 (36%)	53 (9%)	56 (9%)	79 (13%)	230 (38%)	12 (2%)	13 (2%)
Skin conditions (*n* = 200)	96 (48%)	35 (18%)	38 (19%)	41 (20%)	30 (15%)	3 (2%)	4 (2%)

Table excludes those who indicated that monitoring did not show a problem. The 12 health aspects (from top): blood pressure, blood sugar, body composition, cholesterol, mental/cognitive health, eyes/eyesight, diet, fitness/exercise, hearing, heart rate/pulse, menstrual cycle/periods and skin conditions

*GP*  General practitioner, *HP*  Health professional, *GP*  General practitioner, *HP*  Health professional

*N (%)*

**Fig. 3:**
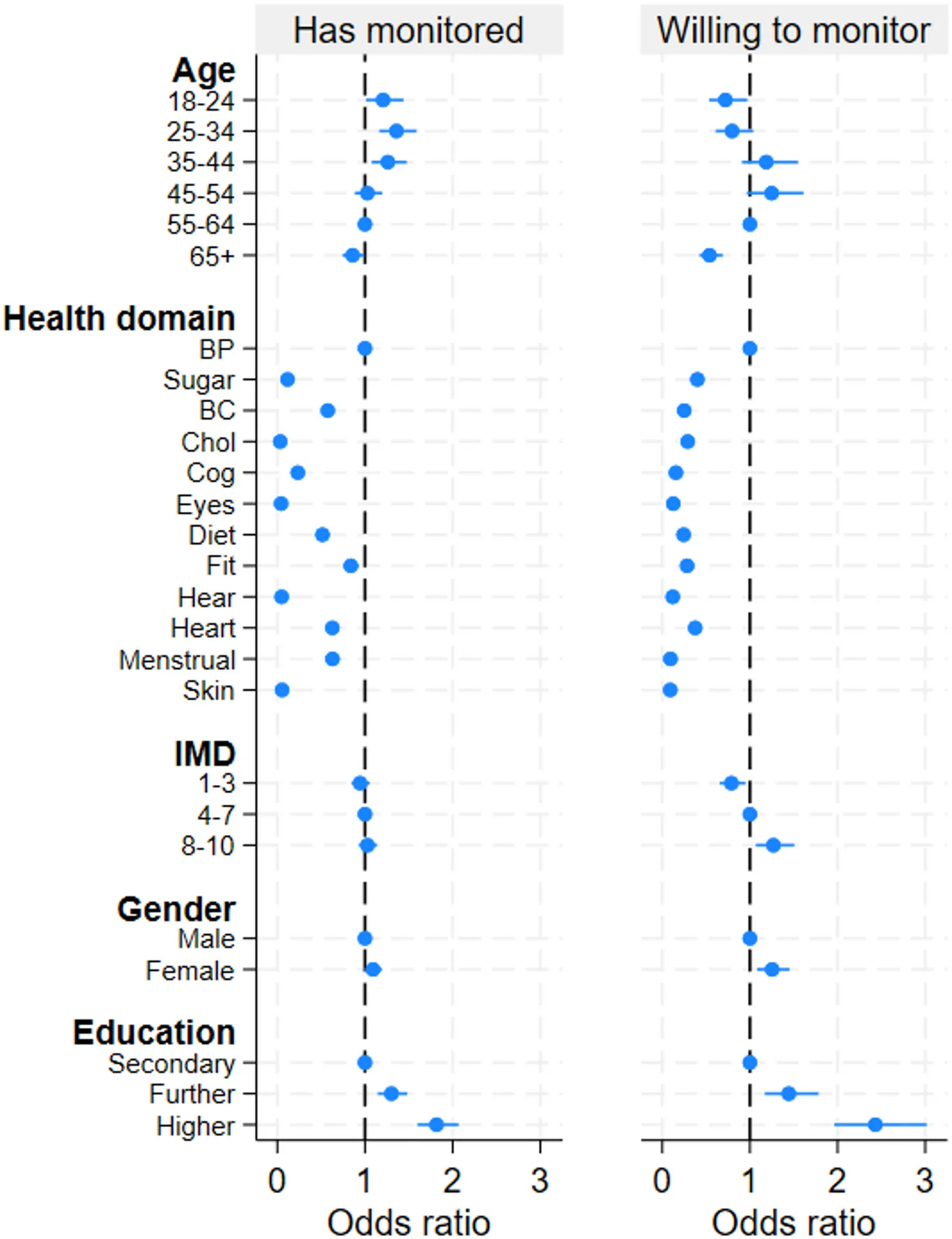
Odds ratios and 95% confidence intervals for ever self-monitoring (left panel) and willing to self-monitor (right panel) health by sociodemographic characteristics and health aspect. The 12 health aspects: BP=blood pressure, Sugar=Blood Sugar, BC=Body Composition, Chol=Cholesterol, Cog=Mental/Cognitive Health, Eyes=Eyesight, Diet, Fit=Fitness/Exercise, Hear=Hearing, Heart=Heart Rate/Pulse, Menstrual=Menstrual Cycle/Periods and Skin=Skin Conditions. IMD=Index of Multiple Deprivation. Education= highest level of education (secondary school, further education but not university degree level, higher education at university degree level)

Figure 3 shows that those with greater levels of education were more likely to have self-monitored compared to those with secondary education (further education OR: 1.30, 95% CI: 1.15, 1.48; higher OR: 1.82; 95% CI: 1.60, 2.07), and compared to respondents aged 55–64, respondents aged ≤ 44 were more likely to have self-monitored (18–24 OR: 1.21, 95% CI: 1.01, 1.44; 25–34 OR: 1.36, 95% CI: 1.16, 1.59; 35–44 OR: 1.26, 95% CI: 1.08, 1.47). Confidence intervals included 1 for gender (OR: 1.09, 95% CI: 1.00, 1.19).

Females were more likely than males to be willing to self-monitor (OR: 1.25, 95% CI: 1.08, 1.45, Fig. 3). Compared to respondents with secondary education, respondents with further and higher education were more likely to be willing to self-monitor (further education OR: 1.44, 95% CI: 1.17, 1.78; higher OR: 2.43, 95% CI: 1.96, 3.01). The youngest (18–24) and oldest (65+) respondents were less likely to be willing to self-monitor compared to respondents aged 55–64 (18–24 OR: 0.72, 95% CI: 0.54, 0.97; 65 + OR:0.54, 95% CI: 0.42, 0.69). Respondents in the most deprived neighbourhoods were less likely to be willing, and respondents in the least deprived areas were more likely to be willing to self-monitor compared to those in areas with middle levels of deprivation (most deprived OR: 0.79, 95% CI: 0.66, 0.95; least deprived OR: 1.27, 95% CI: 1.07, 1.51).

The majority (78%) of respondents were willing for results to be automatically sent to a GP, whereas only one third were willing to pay for an app or other equipment if required (Supplementary Fig. 1). Compared to males, females were less likely to be willing to pay (OR: 0.82, 95% CI: 0.72, 0.95, Supplementary Fig. 2) and younger adults were less likely to be willing to pay compared to older adults (18–24 OR: 0.66, 95% CI: 0.50, 0.87; 25–34 OR: 0.75, 95% CI: 0.58, 0.96). Respondents with further and higher levels of education were more likely to be willing to pay compared to those with secondary education (further OR: 1.25, 95% CI: 1.02, 1.53; higher OR: 1.57, 95% CI: 1.29, 1.93).

If self-monitoring identified a problem, the most preferred action for all age groups was contacting one’s GP followed by making changes to one’s lifestyle (Supplementary Table S3). The proportion of respondents preferring to contact one’s GP increased with age and the proportion preferring to seek online advice decreased with age.

### Perceived benefits and challenges of self-monitoring

Table 3 displays the main reported benefits and problems. Benefits fell into five broad categories: enhanced awareness of health, greater control over health and symptoms, changed engagement with healthcare providers, easy access to health information, and psychologically beneficial. Main problems identified were psychological (e.g., anxiety, obsessive behaviours); behavioural (e.g., around adherence, inaction); potential health outcomes being impacted (e.g., by a lack of guidance and professional support); and technical concerns (e.g., accuracy/need of equipment).

**Table 3:** Categories and subcategories for main benefits of, and problems with, self-monitoring of health

Categories	Subcategories
Benefits	
Enhanced awareness of health status, symptoms and changes in health status and/or symptoms	Earlier identification of a problem
	Earlier diagnosis
	Alert to take action sooner
	An understanding of whether health was changing
	A way to observe trends in health over time
	Ability to track the progress of interventions
	Insights into the impact of lifestyle on health
	Tools for mapping symptoms to behaviours
Greater control over health and symptoms	Allows for regular testing
	Identifying and reducing risks
	Preventing problems
	Providing a record of health status over time
	Ability to maintain better health
Changed engagement with healthcare providers	Sharing of data
	Avoiding an unnecessary medical appointment
	Knowing when it is and is not necessary to seek medical care
	Saving NHS resources
Easy access to health information	Home-based
	Convenient
	Does not require an appointment
	Immediate results
Psychologically beneficial	Providing reassurance and peace of mind
	A motivation to make change and meet goals
	Empowering of self-care
	Encouraging of self-discipline
	Improving self-confidence
	Fostering self-accountability
	Less anxiety provoking than seeing a medical professional
Problems	
Psychological impacts	Cause of anxiety, stress, and hypochondriasis
	The individual becoming overly reliant on and obsessed with self-monitoring
	Self-monitoring being demotivating if goals are not met
	Undesirable results and implications are disregarded
	Results are not trusted
	Individuals lack self-confidence when using equipment and interpreting results
Behavioural factors	Adherence to monitoring
	Monitoring incorrectly and/or inconsistently
	Finding it annoying
	Finding time to do it
	Inaction following monitoring
	Finding it physically uncomfortable
Potential health outcomes being impacted	Lack of guidance and professional support
	Misinterpretation of results
	Missing something important
	Taking incorrect actions following monitoring
	Incorrectly recording data
	Creating a delay in help seeking or diagnosis of a condition
	Doctors don’t take the data seriously
Technical concerns	Accuracy of the equipment and the data
	Equipment failures
	Data privacy/security
	Need for, and cost of, monitoring equipment

## Discussion

### Main finding of this study

This study examined the experiences and opinions of a broadly representative sample of 4270 UK adults concerning self-monitoring of health and personal wellness. The majority of respondents had self-monitored and/or would be willing to do so if recommended by the NHS and were willing for results to be sent to their GP automatically. The proportion of respondents with experience of self-monitoring was generally similar for health as it was for personal wellness; however, the proportion who would be willing to self-monitor if recommended by the NHS was higher for medical than personal wellness. Main benefits included enhanced awareness and control of health; easier access to health information; more balanced engagement with healthcare providers; and psychological support (e.g., reassurance, motivation). Main problems included psychological factors (e.g., anxiety, obsessive behaviours); behavioural factors (e.g., non-adherence, inaction); health outcomes being impacted by a lack of guidance and professional support; and technical concerns around accuracy/needing equipment). Despite a willingness to self-monitor, only one third of respondents were willing to pay for equipment to do so. There were sociodemographic differences in willingness to self-monitor with males, older adults, those living in areas of high deprivation, and those with lower education less likely to be willing to monitor their health.

### What is already known on this topic

Consistent with previous research [11], the current findings demonstrate: (i) a high interest among the general population to monitor their health if recommended by the NHS, and (ii) the public’s openness to have results automatically sent to their GP. The observed sociodemographic differences may be due to differential access to technologies limiting opportunities to engage with self-monitoring and differences in digital health literacy restricting the ability to successfully use self-monitoring tools; indeed, digital health literacy is greater for those with higher education and lower for older adults [15].

Self-monitoring was perceived to be beneficial not only because of the information it provided, but also because it motivated behaviour change, and empowered individuals to maintain healthy behaviours, which aligns with benefits previously identified by GPs [16]. The importance of these aspects must not be underestimated when it comes to health promotion and disease prevention. Many open-ended responses reported the benefit of having ‘evidence’ to take to the doctor; however, a sizeable proportion of respondents indicated they did not act when a problem was identified. It is possible that some members of the public do not consider problems identified to be of sufficient severity to warrant action [17]. Additionally, challenges in accessing health services post-monitoring [18] and limited opportunities for lifestyle changes (e.g., associated with social determinants of health) [19] could hinder action being taken. This study found trust in results was similar for healthcare provider recommended and self-initiated monitoring, but proportionally more respondents took action when monitoring was recommended by a health professional. Reasons for this difference are unknown, but it is possible that self-monitoring recommended by a health professional included follow-up contact and additional tailored advice to support action being taken. Previous systematic reviews (e.g., for diet [20], blood pressure [21]) found combining self-monitoring with tailored support (e.g., education/professional support) to be more effective in improving health outcomes than self-monitoring alone. Thus, there is a need to establish how self-monitoring can be strengthened to maximise action. A solution may be to have NHS ‘approved’ self-monitoring apps to increase trust in, and validity of, the results.

### What this study adds

Incorporating self-monitoring results into NHS records presents an opportunity to identify health concerns early and aligns with government initiatives for preventative action to promote population health [5, 22]. Self-monitoring supports proactive management of health remotely however, challenges may arise if results are unreliable or misunderstood by users and could potentially lead to workload/capacity concerns if users seek follow-up professional support. A systematic review found healthcare providers perceived self-monitoring data to be valuable for supporting patients, healthcare providers, and patient-provider relationships but found limited engagement with this data in clinical practice [23]. Thus, there is uncertainty in what types of self-monitoring data might be practical for healthcare providers to use in clinical workflows. Respondents were willing to self-monitor, if recommended by the NHS, more so for health conditions than personal wellness. While the current study found high levels of trust in the results among those who self-monitor, there is a need to explore healthcare provider perspectives. The preferred action by respondents if self-monitoring identified a problem was to initiate contact with their GP, rather than waiting to be contacted. This approach could potentially add pressure on GP availability although this could be alleviated through initiatives outlined in the 10 Year Health plan for England such as an NHS App to allow for instant advice and access to support [5]. While there are potential benefits of self-monitoring, there is also the potential for inequalities to be exacerbated due to differential access/use across socioeconomic characteristics, differences in digital literacy, and variations in outcomes [24]. The lack of standardization and validation of wearable devices (i.e., not equivalent to clinical-grade medical devices) makes it difficult for GPs to interpret the data. This study identified socioeconomic differences in willingness to self-monitor. As such, approaches that promote inclusive use of self-monitoring such as educational programmes, provider assistance and accessible support present opportunities for wider access and use of self-monitoring of health [25].

Examples of unanswered questions include:

- How can engagement in self-monitoring of health be enhanced?
- Why is trust in results for some health aspects lower than others?
- Why do some individuals not act when a problem is identified?
- Are there individuals for whom self-monitoring will lead to negative psychological outcomes and who are they?

### Limitations of this study

The survey contained just nine questions, and it did not gather data on the frequency of monitoring, whether self-monitoring resulted in any health improvements, or the motivations for self-monitoring. It is possible that including ‘condition’ in the phrasing of some of the questions might have conflated medical monitoring with personal wellness monitoring: individuals may opt to monitor health for medical reasons (e.g., if they have a specific condition such as diabetes, or risk factor(s) for a disease) that may require ongoing engagement with healthcare providers, or for personal wellness reasons (e.g., monitoring behaviour such as fitness/exercise) independent of the healthcare service. While the survey included examples of self-monitoring for both medical and wellness purposes, only a small number of illustrative health aspects were included and other aspects of health (e.g., sleep, bowel, urine etc.) present opportunities for self-monitoring. Respondents had the option to indicate ‘Other’ health aspects they had self-monitored though few did so. Due to question phrasing and to keep the survey to a reasonable length, we are unable to determine whether there were differences in perceived benefits and problems of self-monitoring when monitoring was recommended by a healthcare professional or self-initiated.

A limitation of the study is that the online survey may have been completed by individuals with high digital literacy. To keep the survey to a reasonable length, limited contextual information was provided on what would be involved in self-monitoring and we did not ask respondents how they would expect to self-monitor each of the health aspects. Therefore, it is possible that respondents, particularly those with lower health literacy, did not have sufficient information to provide informed responses. Further details on the type of monitoring, and whether it is likely to involve pain and/or cost for the user may have led to different findings.

Online surveys of health are common e.g., Health Insight Survey conducted by the office for National Statistics and funded by NHS England [26]. However, a concern about online health surveys is acquiescence bias i.e., the tendency for respondents to agree with statements, often driven by a desire to be polite [27]. In the present study, this could result in an inflated willingness to self-monitor. While acquiescence bias cannot be ruled out entirely, this is not supported by the reduced willingness to self-monitor personal wellness compared to health conditions, or the list of perceived problems in the open-ended question.

Nevertheless, online surveys are quick to administer to a large and geographically diverse sample. Strengths included gathering information from a broadly representative sample population, examination of sociodemographic influences on willingness to self-monitor health, and incorporation of feedback from members of the public to ensure the survey was understandable and of reasonable length.

## Conclusions

Self-monitoring has the potential to transform health. The findings of this study highlight the UK public’s high level of use and trust in self-monitor. There was a willingness to self-monitor, if recommended by the GP and have their results to be sent directly to their GP, particularly for medical health conditions. These results align with ongoing government initiatives to transform NHS care through preventative, community-based, action. However, the findings also highlight socioeconomic differences in self-monitoring which has the potential to exacerbate inequalities. Future research is needed to address how engagement of self-monitoring can be enhanced equitably and used effectively alongside clinical care to promote better health.

## Supplementary Information

Supplementary Material 1.

## Data Availability

Data is available from the corresponding author on reasonable request.

## Abbreviations

CI: Confidence interval
IMD: Index of multiple deprivation
NHS: National health Service
OR: Odds ratio
UK: United Kingdom of Great Britain and Northern Ireland
