# Supplementary material for: Use, trustworthiness and willingness to self-monitor health and personal wellness: a UK population survey
Source: Arch Public Health. 2026 Aug 13;84:240. doi: 10.1186/s13690-026-02035-6 (PMC13749996; doi:10.1186/s13690-026-02035-6)
Supplement: Supplementary file 1 [file 13690_2026_2035_MOESM1_ESM.docx]

**Supplementary materials**

**Table S1. Online survey questions. Response options shown in italics.**

Self-monitoring means checking/monitoring your health on your own, even if it was recommended to you by someone else. This could involve, for example, using a mobile app/website, wearable device, digital monitor, questionnaire or diary, and taking repeated measurements over weeks or months.

1. **Have you ever self-monitored any of the following aspects of your health? Please select all that apply.**

*Blood pressure / Blood sugar / Body composition / Cholesterol / Diet / Eyesight / Fitness/exercise / Hearing / Menstrual cycle/periods / Mental and cognitive health / Pulse/heart rate / Skin conditions / Other / None / Prefer not to say*

2. **In general, to what extent, if at all did you trust the results when you self-monitored your:** [Applies to each condition monitored in question 1]

*A lot / A little / Not very much / Not at all / Prefer not to say*

3. **If the monitoring showed a problem or concern, did you act on the results? Please select all the options that apply for each condition.** [Applies to each condition monitored in question 1]

*N/A- monitoring did not show a problem / Discussed with GP / Discussed with other health professional /Sought advice online / Changed my life/lifestyle / Did not take any action / Don’t know / Prefer not to say*

4. **You said that you self-monitored the following aspects of your health. For which, if any, was self-monitoring recommended to you by a health professional such as your GP? Please select all that apply.**

*[Personalised from the responses to question 1]*

5. **What were the main benefits, or what do you think would be the main benefits, of self-monitoring your health? Please type your answers in the box below, providing as much details as possible).**
6. **What were the main problems, or what do you think would be the main problems with self-monitoring your help? Please type your answers in the box below, providing as much details as possible.**
7. **If it was recommended by the NHS in the future, which, if any of the following would you be willing to self-monitor (or continue to self-monitor)? Please select all that apply.**

*Blood pressure / Blood sugar / Body composition / Cholesterol / Diet / Eyesight / Fitness/exercise / Hearing / Menstrual cycle/periods / Mental and cognitive health / Pulse/heart rate / Skin conditions / Other / None / Prefer not to say*

8. **In general, if health self-monitoring was recommended by the NHS, to what extent, if at all, would you be willing:** [Only if willing to monitor at least one condition in question 6]

**8a. For the results of your self-monitoring to be automatically sent to your GP or other health professional**

*Very willing / Fairly Willing / Neither willing or unwilling / Fairly unwilling / Very unwilling / Don’t know / Prefer not to say*

**8b. To pay for a mobile app or other equipment required to self-monitor your health (assuming a similar cost to an NHS prescription).**

*Very willing / Fairly Willing / Neither willing or unwilling / Fairly unwilling / Very unwilling / Don’t know / Prefer not to say*

9. **In general, if health self-monitoring identified a problem, what would be your preferred next steps? Please select up to three options.** *[Only if willing for at least one condition in question 6]*

*Wait for my GP to contact me to discuss / Wait for a health professional who is not my GP to contact me to discuss e.g., pharmacist / Contact my GP to discuss / Contact a health professional who is not my GP to discuss e.g., pharmacist / Seek online advice / Make changes to my life/lifestyle / Take no action / Don’t know / Prefer not to say*

**Table S2. Proportion of respondents who indicated monitoring did not show a problem by health aspect monitored.**

| Health aspect | Number who had monitored | Monitoring did not show a problem or concern |
| --- | --- | --- |
| Blood Pressure | 1733 | 722 (42%) |
| Blood Sugar | 443 | 158 (36%) |
| Body Composition | 1306 | 457 (35%) |
| Cholesterol | 149 | 40 (27%) |
| Diet | 1227 | 436 (36%) |
| Eyesight | 194 | 61 (31%) |
| Fitness/exercise | 1591 | 820 (52%) |
| Hearing | 214 | 80 (37%) |
| Mental/cognitive health | 742 | 160 (22) |
| Menstrual cycle | 738 | 396 (54%) |
| Pulse/heart rate | 1369 | 768 (56%) |
| Skin conditions | 235 | 35 (15%) |

**Table 3. Preferred courses of action by age if self-monitoring identified a problem or concern among respondents who were willing to monitor at least one aspect of their health. N=3678 participants who were willing to monitor at least one aspect of their health. Participants invited to select up to 3 options.**

| Action | Age, n(%) | | | | | | |
| --- | --- | --- | --- | --- | --- | --- | --- |
|  | 18-24 | 25-34 | 35-44 | 45-54 | 55-64 | 65+ | Total |
| Contact my GP to discuss | 231 (64%) | 414 (66%) | 412 (68%) | 490 (72%) | 429 (75%) | 669 (80%) | 2645 (72%) |
| Make changes to my life/lifestyle | 158 (44%) | 300 (48%) | 320 (53%) | 285 (42%) | 239 (42%) | 296 (35%) | 1598 (43%) |
| Seek online advice | 132 (37%) | 205 (33%) | 171 (28%) | 164 (24%) | 111 (19%) | 151 (18%) | 934 (25%) |
| Wait for my GP to contact me to discuss | 75 (21%) | 161 (26%) | 141 (23%) | 157 (23%) | 133 (23%) | 164 (20%) | 831 (23%) |
| Contact a health professional who is not my GP to discuss | 61 (17%) | 124 (20%) | 122 (20%) | 135 (20%) | 120 (21%) | 192 (23%) | 754 (21%) |
| Wait for a health professional who is not my GP to contact me to discuss | 29 (8%) | 64 (10%) | 55 (9%) | 67 (10%) | 64 (11%) | 61 (7%) | 340 (9%) |
| Take no action | 12 (3%) | 12 (2%) | 10 (2%) | 9 (1%) | 4 (1%) | 3 (<1%) | 50 (1%) |
| Don't know | 8 (2%) | 18 (3%) | 20 (3%) | 19 (3%) | 11 (2%) | 10 (1%) | 86 (2%) |
| Prefer not to say | 3 (1%) | 7 (1%) | 2 (<1%) | 2 (<1%) | 1 (<1%) | 0 (0%) | 15 (<1%) |
| *GP = General practitioner* |  |  |  |  |  |  |  |

**Figure S1. Willingness to pay for an app or other equipment required to self-monitor by age, gender, index of multiple deprivation (IMD), and education.**

**Figure S2. Association between sociodemographic characteristics and willingness to pay for an app or other equipment required to self-monitor.**
